# Dr. Thomas Soars Johnson

**Published:** 1910-09-10

**Authors:** 


### OBITUARY.

The death is announced of Dr. Thomas Soars Johnson, coroner of Canterbury for nearly thirty years. While holding an inquest last week at the municipal offices he was seized with apoplexy and never fully regained consciousness. He was a St. Andrews student, and for some years in private practice in the North before he came to Canterbury.

